# Comparison of National Institutes of Health-Chronic Prostatitis Symptom Index with International Index of Erectile Function 5 in Men with Chronic Prostatitis/Chronic Pelvic Pain Syndrome: A Large Cross-Sectional Study in China

**DOI:** 10.1155/2015/560239

**Published:** 2015-07-27

**Authors:** Jingjing Gao, Pan Gao, Zongyao Hao, Zengrong Zhou, Jihong Liu, Hongjun Li, Junping Xing, Zhansong Zhou, Chunhua Deng, Liwen Deng, Qiang Wei, Xiansheng Zhang, Jun Zhou, Song Fan, Sheng Tai, Chen Yang, Kai Shi, Yuanyuan Huang, Zhangqun Ye, Chaozhao Liang

**Affiliations:** ^1^Department of Urology, The First Affiliated Hospital of Anhui Medical University, Hefei, Anhui 230022, China; ^2^Department of Urology, The Tongji Hospital of Huazhong University of Science and Technology, Wuhan, Hubei 430000, China; ^3^Department of Urology, The Beijing Xiehe Hospital, Beijing 100000, China; ^4^Department of Urology, The First Affiliated Hospital of Xi'an Jiaotong University, Xi'an, Shanxi 710000, China; ^5^Department of Urology, The Southwest Hospital of Third Military Medical University, Chongqing 404100, China; ^6^Department of Urology, The First Affiliated Hospital of Zhongshan University, Guangzhou, Guangdong 510000, China; ^7^Department of Urology, The Huaxi Hospital of Sichuan University, Chengdu, Sichuan 610000, China

## Abstract

The purpose of the study is to evaluate the relationship between NIH-CPSI and IIEF-5 in Chinese men with CP/CPPS. A large cross-sectional and multicenter survey was conducted from July 2012 to January 2014. Men were recruited from urology clinics which were located at the five cities in China. All men participated in the survey by completing a verbal questionnaire (consisted of sociodemographics, past medical history, sexual history, and self-estimated scales). The results showed that 1,280 men completed the survey. Based on the CP/CPPS definition, a total of 801 men were diagnosed as having CP/CPPS. Men with CP/CPPS reported higher scores of NIH-CPSI and lower scores of IIEF-5 than men without CP/CPPS. NIH-CPSI scores were significantly negatively correlated with IIEF-5 scores. The total scores of NIH-CPSI were significantly more strongly correlated with question 5 than other questions of IIEF-5. The total scores of IIEF-5 were significantly more strongly correlated with pain symptoms scores of NIH-CPSI. Strongest correlation was found between QoL impact and question 5 of IIEF-5. The findings suggested that NIH-CPSI scores were significantly negatively correlated with IIEF-5 scores. Strongest correlation was found between QoL impact and question 5 of IIEF-5.

## 1. Introduction

Chronic prostatitis/chronic pelvic pain syndrome (CP/CPPS) is a common yet poorly understood condition, with severe impact on the quality of life (QoL) of diagnosed patients, especially on sexual function [1, 2]. The prevalence of CP/CPPS was estimated between 2.2% and 13.8% [3]. Similarly, a population-based sample of Chinese men has shown that the prevalence of CP/CPPS-like symptoms is 4.5% in China [4].

Previous studies have showed that CP/CPPS (evaluated by the National Institutes of Health-Chronic Prostatitis Symptom Index [NIH-CPSI]) is a common urological condition that includes pelvic/genital pain and urinary dysfunction, same for any other chronic pain conditions, and many patients with CP/CPPS reported reduced quality of life [5]. Schaeffer et al. [6] examined 488 men with the clinical diagnosis of CP/CPPS in the NIH chronic prostatitis cohort. They found noticeable increased NIH-CPSI scores in patients with localized tenderness on physical examination. Supporting their findings, Marszalek et al. [7] also discovered such an association with regard to the perineum, testicles, penis, and suprapubic area as a site of self-reported discomfort. Similarly, another study conducted by Turner et al. [8] showed a close correlation between pelvic pain scores and impaired on quality of life in the 357 men with prostatitis which suggested that male pelvic pain and associated symptoms may have a significant negative impact on health-related QoL. In a study that evaluated the management of CP/CPPS patients in primary care settings, results showed that worse QoL is associated with greater pain and urinary symptoms and that pelvic pain is associated with worse QoL than urinary symptoms [9].

In addition, while the symptoms of CP/CPPS are not life-threatening, a significant proportion of men reported that a high percentage of men with CP/CPPS suffer from some form of sexual dysfunction including ED, premature ejaculation, and painful ejaculation [10]. From a study that evaluated the prevalence, relevant factors, and effects of sexual dysfunction in primary care referral populations, sexual disorders (self-reported ED or ejaculatory difficulty or both) were reported by 72% of patients with CP/CPPS [11]. Similarly, in another questionnaire-based study (*n* = 1,765), Vienna men reported that NIH-CPSI score was the risk factor of ED (odds = 8.3) [10]. Unfortunately, while the NIH-CPSI contains items on pain, urination, and quality of life, it totally omits questions with regard to sexual dysfunction [12]. The Chinese version of International Index of Erectile Function 5 (IIEF-5), a widely used, validated, self-administered questionnaire, has been demonstrated to be highly sensitive and specific to ED [13]; it contains five questions, and question 5 is about the sexual satisfaction during sexual intercourse.

With the improvement of the standard of living, people paid more attention to quality of life issues, particularly sexual dysfunction. Although several previous studies have shown that CP/CPPS has a negative influence on erectile function [14], the relationship between CP/CPPS and ED still remains unclear, particularly in China. The relationship between NIH-CPSI and IIEF-5 in a large population study has not been reported. Hence, in this study, we aimed to investigate the relationship between NIH-CPSI and IIEF-5 in Chinese men with CP/CPPS.

## 2. Subjects and Methods

### 2.1. Subjects

A large cross-sectional and multicenter survey was conducted from July 2012 to January 2014. Based on the stratified sampling, several different cities (including Beijing, Guangzhou, Hubei, Shanxi, and Anhui) were selected randomly to represent the northern, southern, middle, western, and eastern parts of China. Male participants were recruited from men seeking care for CP/CPPS in urologic clinics.

For inclusion, men had to be aged ≥18 years and have had a heterosexual, stable, and monogamous sexual relationship for at least 6 months. In addition, because some subjective questions were asked in our study, eligible men should be able to comprehend and speak Chinese. Each subject's medical and sexual history was carefully evaluated by an experienced clinician. Men on medications that could affect their erectile function were excluded. This study was evaluated and approved by Anhui Medical University Research Subject Review Board.

### 2.2. Study Design and Procedure

Prior to study enrollment, all participants were informed about this survey. Eligible patients were asked to provide written consent. In addition, a presurvey (*N* = 30) was given to a small sample of subjects to modify the original designed items to ensure that the questionnaire was comprehensive and easily understood.

All men participated in the survey by completing a verbal questionnaire, which consisted of sociodemographics (e.g., weight, height, age, marital status, and education level), past medical history, sexual history, and self-estimated scales (e.g., the Chinese version of IIEF-5 and NIH-CPSI).

The definition of CP/CPPS was used as described in the NIH consensus. Assessment of NIH-CPSI is a reliable, convenient, self-administered index that is widely used across scientific research and clinical studies (including pain symptoms [total of items 1–4], urinary symptoms [total of items 5 and 6], and quality of life impact [total of items 7–9]). The Chinese version of NIH-CPSI was widely used in the previous studies in China. Based on the total of items 1–9, the severity of CP/CPPS was classified as mild (10–14 points), moderate (15–29 points), or severe (>30 points). ED was measured by the IIEF-5 instrument (Table 1), in its Chinese version. This instrument contained 5 questions, each of which was graded on a scale from 0 to 5 points. An IIEF-5 score ≥ 22 indicated normal erectile function and <22 indicated ED. The reliability of NIH-CPSI and IIEF-5 in this study was 0.82 and 0.79, respectively, demonstrating acceptable internal reliability.

### 2.3. Statistical Analysis

Data analyses were carried out with SPSS version 13.0 software (SPSS Inc., Chicago, IL, USA). Descriptive statistics were used to summarize the characteristics of the subjects. Data were expressed as the mean ± standard deviation or number (percentage) when appropriate. Chi-square and Mann-Whitney *U* tests were used for intergroup comparisons. Correlations between the outcomes of the IIEF-5 and NIH-CPSI in men with CPPS were assessed using partial correlations analysis, and all data were adjusted for age because of the sexual dysfunctions known to be age-dependent phenomena. For all tests, *P* < 0.05 was considered statistically significant.

## 3. Results

Of 1,672 men who met inclusion criteria, 1,280 men completed the survey, with a response rate of 76.56%. Men discontinued the study (*n* = 392, 23.44%) for the following reasons: “withdrawal of consent” (*n* = 215, 12.86%), “incomplete information” (*n* = 110, 6.58%), and “other reasons” (*n* = 67, 4.01%). Their mean ages and BMI scores were 34.50 ± 9.20 years and 24.36 ± 1.70 kg/m^2^, respectively. Based on the CP/CPPS definition, a total of 801 men were diagnosed as having CP/CPPS. The incidence of CP/CPPS in all subjects was 62.58%. There was no significant difference between CP/CPPS and control groups, with respect to demographic information (e.g., age, BMI scores, and educational status) (*P* < 0.001 for all). Detailed demographic characteristics for all subjects were summarized in Table 2.

Results from Table 3 showed that men with CP/CPPS reported higher scores of NIH-CPSI and lower scores of IIEF-5 than men without CP/CPPS (*P* < 0.001 for all). The mean total scores of NIH-CPSI and IIEF-5 in CP/CPPS groups were 31.23 ± 9.86 and 17.70 ± 3.25, respectively, while those in control group were 1.89 ± 1.17 and 23.23 ± 3.84. In addition, for the subdomain of NIH-CPSI (including items of pain, urinary symptoms, or QoL impact), a significant difference was observed in men with and without CP/CPPS (*P* < 0.001 for all). Men with CP/CPPS reported worse pain and urinary symptoms and QoL impact than men without CP/CPPS. Similarly, men with CP/CPPS complained of worse erectile function than men without CP/CPPS. The mean subdomain scores of IIEF-5 in CP/CPPS group were significantly lower than those in control group (*P* < 0.001 for all).

Several correlations were presented among the outcomes of NIH-CPSI and IIEF-5 in CP/CPPS group (Table 4). NIH-CPSI scores were significantly negatively correlated with IIEF-5 scores (*P* < 0.001 for all). For the total scores of NIH-CPSI, they were significantly more strongly correlated with question 5 than other questions of IIEF-5 (Adjusted *r* = −0.70, *P* < 0.001). In addition, the total scores of IIEF-5 were significantly most strongly correlated with pain symptoms scores of NIH-CPSI (Adjusted *r* = −0.70, *P* < 0.001). Of the correlation between subdomains of NIH-CPSI and IIEF-5, the strongest correlation was found between QoL impact and question 5 of IIEF-5 (Adjusted *r* = −0.74, *P* < 0.001).

## 4. Discussion

This is a large population-based study to systematically evaluate the NIH-CPSI scores and IIEF-5 scores and to find their possible contact details in Chinese people and further to explore the influence of CP/CPPS on sexual dysfunction, especially on ED. In our study, male participants were located at the cities which were selected randomly to represent the northern, southern, middle, western, and eastern parts of China (including Beijing, Guangzhou, Hubei, Shanxi, and Anhui). It is not surprising that comparing the contact details between NIH-CPSI and IIEF-5 within the greater population has a highly significant and might provide a framework for understanding of the association between ED and CP/CPPS in China.

The results from our study showed that men with CP/CPPS presented higher scores of NIH-CPSI and lower scores of IIEF-5. Men with CP/CPPS also reported worse pain symptoms, urinary symptoms, QoL impact, and worse erectile function than men without CP/CPPS. In addition, negative relationship between NIH-CPSI score and IIEF-5 score was found in men with CP/CPPS. Based on the Adjusted *r* values, these negative relationships were stronger between QoL impact and question 5 of IIEF-5 (the sexual satisfaction during sexual intercourse).

From the previous study, we know that pain is the most significant symptom of CP/CPPS [12] and pain symptoms are heterogeneous, although the most commonly reported symptom is pain during or following ejaculation [15, 16]. Although the most common locations of pain are perineal, testicular, and penile areas, the pain may also radiate to various areas of the pelvis, causing pain in the lower abdomen, upper legs, and lower back. Some kinds of activities often aggravate the pain, for example, sitting, physical activities, and particularly sexual activity [12]. It should be noted that there is strong evidence showing that pain catastrophizing has an influence on symptom severity [17]. Furthermore, what we need to beer in mind is that men may also suffer urinary symptoms, including dysuria (painful urination), frequent urination, and the sensation of incomplete urination, and these urinary symptoms have a negative impact on quality of life [12]. More importantly, our findings in this research are further confirmed in the aforementioned studies.

A study conducted by Lee et al. [18] found that sexual dysfunction, especially the combination of ED and ejaculatory difficulties, was associated with substantial reductions in QoL. Another study by Zhao et al. [1] found that ED is obviously quite common among the men with CP/CPPS. Similarly, in the present study, we found that men with CP/CPPS complained of worse erectile function than men without CP/CPPS. In a cross-sectional survey of 15,000 Chinese men, Hao et al. [19] found that the prevalence of ED was higher in the prostatitis population than in the general population with either self-reported or IIEF-5 score assessment. In their study, among 771 men with prostatitis-like symptoms, ED prevalence was 39.3% and 30.1%, assessed by self-report and IIEF-5 score, respectively, and among 370 men suffering from chronic prostatitis, ED prevalence was 40.5% and 35.1%, respectively. Their data provide further evidence that ED is related to prostatitis and that the prevalence is higher in the CP and prostatitis-like symptoms group than in the general group. In their study, the relationships between ED and CP/CPPS symptoms were obvious. Similarly, the information from Magri et al. [11] showed the direct relation between the severity of CP symptoms and ED frequency and severity of ED. From the results of their research, the frequency of ED was showing higher total scores of two administered questionnaires, that is, the NIH-CPSI and the International Prostate Symptoms Score (IPSS). Furthermore, several previous studies have shown that CP/CPPS has a negative effect on erectile function [7, 18, 20, 21], and a cross-sectional survey in Singapore conducted by Tan et al. [22] showed that participants with CP/CPPS had worse erectile function (*P* < 0.003) than those without CP/CPPS. Interestingly and importantly, results from our study further confirmed their findings. In our study, negative associations between IIEF-5 scores and NIH-CPSI scores were obvious in men with complaints of CP/CPPS.

Furthermore, negative relationships between NIH-CPSI score and IIEF-5 score were observed in men with CP/CPPS. From the previous studies, we found that men with both erectile and ejaculatory difficulties had significantly greater NIH-CPSI total scores and worse QoL subscores [18] and according to the data from some other authors we know that CP/CPPS impairs the overall quality of life and causes erectile dysfunction [23]. Therefore, we speculated that the negative QoL impact from prostatic symptoms might be correlated with the decreased sexual satisfaction of patients with CP/CPPS, and it may affect the overall quality of their intimate relationship with their sexual partners, and this might be associated with the etiology of ED. Similarly, some articles have reported that people who have a certain degree of sexual satisfaction have noticeably better quality of life than those who reported no sexual satisfaction [24]. In particular, our finding showed that QoL impact of NIH-CPSI was most strongly correlated with question 5 (the sexual satisfaction during sexual intercourse) of IIEF-5. However, the above association between the QoL impact and sexual satisfaction might be impacted by more factors, and future researches are needed to clear the relations between CP/CPPS and ED.

Our study provides a framework for understanding the relationships between NIH-CPSI and IIEF-5 in Chinese men with CP/CPPS. However, several limitations of this survey should be considered. First, although the subdomain of NIH-CPSI (including items of pain, urinary symptoms, or QoL impact) was evaluated and compared with total and each question of IIEF-5, we did not compare each question of IIEF-5 in men with CP/CPPS with those in men without CP/CPPS. This is not necessarily a reason of weakness. Second, because this study was a cross-sectional design, the results cannot be interpreted as risk or predictive factors. The statistically significant factors encountered in those analyses can be regarded as associations that need to be further studied in prospective studies. Third, although 1,672 men have been selected, approximately 23.44% discontinued the study. Potential sampling bias should be considered. Finally, because some sensitive and personal questions on sexual history were brought in our study, the participants' bias should also be considered.

## 5. Conclusions

This is a large population-based study to systematically evaluate the contact details between NIH-CPSI and IIEF-5 in men with CP/CPPS in detail in China. We found that men with CP/CPPS presented higher scores of NIH-CPSI and lower scores of IIEF-5. Men with CP/CPPS also reported worse pain symptoms, urinary symptoms, QoL impact, and worse erectile function than men without CP/CPPS. In addition, negative relationships between NIH-CPSI score and IIEF-5 score were found in men with CP/CPPS. The negative relationships between QoL impact and question 5 of IIEF-5 (the sexual satisfaction during sexual intercourse) were strongest. Future researches are needed to clear the relations between CP/CPPS and ED.

## Figures and Tables

**Table 1:** Questions of International Index of Erectile Function 5.

Question	Score
*Q1.* How do you rate your confidence that you could get and keep an erection?	1 = Very low 2 = Low 3 = Moderate 4 = High 5 = Very high

*Q2.* When you had erections with sexual stimulation, how often were your erections hard enough for penetration?	0 = No sexual activity 1 = Almost never or never 2 = A few times (less than half the time) 3 = Sometimes (about half the time) 4 = Most times (more than half the time) 5 = Almost always or always

*Q3.* During sexual intercourse, how often were you able to maintain your erection after you had penetrated (entered) your partner?	0 = Did not attempt intercourse 1 = Almost never or never 2 = A few times (less than half the time) 3 = Sometimes (about half the time) 4 = Most times (more than half the time) 5 = Almost always or always

*Q4.* During sexual intercourse, how difficult was it to maintain your erection to completion of intercourse?	0 = Did not attempt intercourse 1 = Extremely difficult 2 = Very difficult 3 = Difficult 4 = Slightly difficult 5 = Not difficult

*Q5.* When you attempted sexual intercourse, how often was it satisfactory for you?	0 = Did not attempt intercourse 1 = Almost never or never 2 = A few times (less than half the time) 3 = Sometimes (about half the time) 4 = Most times (more than half the time) 5 = Almost always or always

**Table 2:** Demographic information of men in the CPPS and control groups.

Characteristics	All (*N* = 1280)		CPPS (*N* = 801)		Control (*N* = 479)		*P*
Age (years)	34.50 ± 9.20		34.90 ± 9.43		33.84 ± 9.24		**0.81**
BMI (kg/m^2^)	24.36 ± 1.70		24.20 ± 1.68		24.64 ± 1.82		**0.63**
Marital status (*n* %)							**0.96**
Single	233	18.20%	150	18.73%	83	17.33%	
Married	834	65.16%	519	64.79%	315	65.76%	
Separate or divorced	213	16.64%	132	16.48%	81	16.91%	
Educational status (*n* %)							**0.83**
Primary school	536	41.88%	337	42.07%	199	41.54%	
High school	415	32.42%	257	32.08%	158	32.99%	
University graduate	329	25.70%	207	25.84%	122	25.47%	
Occupational status (*n* %)							**0.91**
Student	114	8.91%	72	8.99%	42	8.77%	
Unemployed	70	5.47%	41	5.12%	29	6.05%	
Employed	1040	81.25%	655	81.77%	385	80.38%	
Retired	56	4.38%	33	4.12%	23	4.80%	

CPPS = chronic pelvic pain syndrome; N/A = not applicable.

*P*: difference between CPPS and control groups.

**Table 3:** Outcomes of NIH-CPSI and IIEF-5 in CPPS and control groups.

Characteristics	All subjects (*N* = 1280)	CPPS (*N* = 801)	Control (*N* = 479)	*P*
NIH-CPSI (scores)				
Total scores	20.25 ± 6.52	31.23 ± 9.86	1.89 ± 1.17	**<0.001**
Pain symptoms	10.49 ± 4.47	16.34 ± 5.25	0.72 ± 0.25	**<0.001**
Urinary symptoms	5.27 ± 2.18	8.03 ± 3.13	0.65 ± 0.33	**<0.001**
Quality of life impact	4.49 ± 1.92	6.86 ± 2.24	0.52 ± 0.19	**<0.001**
IIEF-5 (scores)				
Total score	19.77 ± 3.62	17.70 ± 3.25	23.23 ± 3.84	**<0.001**
Q1	3.67 ± 0.82	3.02 ± 1.15	4.75 ± 0.62	**<0.001**
Q2	3.68 ± 1.02	3.16 ± 1.07	4.56 ± 0.78	**<0.001**
Q3	4.13 ± 0.95	3.89 ± 0.72	4.52 ± 1.02	**<0.001**
Q4	4.35 ± 0.67	4.11 ± 0.43	4.74 ± 0.82	**<0.001**
Q5	3.95 ± 0.94	3.52 ± 1.24	4.66 ± 0.75	**<0.001**

Data are expressed as the mean ± standard deviation.

Differences between CPPS and control groups were assessed by Mann-Whitney *U* test.

NIH-CPSI = National Institute of Health-Chronic Prostatitis Symptoms Index; CPPS = chronic pelvic pain syndrome; PE = premature ejaculation; IIEF-5 = International Index of Erectile Dysfunction 5.

*P*: difference between CPPS and control groups.

**Table 4:** Correlation between NIH-CPSI and IIEF-5 in men with CPPS.

	IIEF-5 score											
	Total score		Q1		Q2		Q3		Q4		Q5	
	Adjusted *r*	*P*	Adjusted *r*	*P*	Adjusted *r*	*P*	Adjusted *r*	*P*	Adjusted *r*	*P*	Adjusted *r*	*P*
NIH-CPSI scores												
Total score	−0.65	<0.001	−0.6	<0.001	−0.62	<0.001	−0.61	<0.001	−0.65	<0.001	−0.7	<0.001
Pain symptoms	−0.7	<0.001	−0.65	<0.001	−0.67	<0.001	−0.69	<0.001	−0.68	<0.001	−0.67	<0.001
Urinary symptoms	−0.68	<0.001	−0.62	<0.001	−0.64	<0.001	−0.68	<0.001	−0.66	<0.001	−0.65	<0.001
Quality of life impact	−0.64	<0.001	−0.69	<0.001	−0.62	<0.001	−0.64	<0.001	−0.62	<0.001	−0.74	<0.001

NIH-CPSI = National Institute of Health-Chronic Prostatitis Symptoms Index; CPPS = chronic pelvic pain syndrome; IIEF-5 = International Index of Erectile Dysfunction 5.
